# TiO_2_-KH550 Nanoparticle-Reinforced PVA/xylan Composite Films with Multifunctional Properties

**DOI:** 10.3390/ma11091589

**Published:** 2018-09-02

**Authors:** Xinxin Liu, Xiaofeng Chen, Junli Ren, Chunhui Zhang

**Affiliations:** State Key Laboratory of Pulp and Paper Engineering, School of Light Industry and Engineering, South China University of Technology, Guangzhou 510640, China; lxx19910312@163.com (X.L.); chenxiaofeng9108@163.com (X.C); renjunli@scut.edu.cn (J.R.)

**Keywords:** polyvinyl alcohol/xylan composite films, TiO_2_-KH550 nanoparticle, mechanical property, UV shielding

## Abstract

In order to improve the strength of polyvinyl alcohol (PVA)/xylan composite films and endow them with ultraviolet (UV) shielding ability, TiO_2_-KH550 nanoparticles was synthesized and added into the PVA/xylan matrix. The TiO_2_-KH550 nanoparticle dispersed well in the 0.04% sodium hexametaphosphate (SHMP) solution under ultrasonic and stirring treatments. Investigations on the properties of the films showed that TiO_2_-KH550 had the positive impact on improving the strength, moisture, and oxygen barrier properties of the composite films. The maximum tensile strength (27.3 MPa), the minimum water vapor permeability (2.75 × 10^−11^ g·m^−1^·s^−1^·Pa^−1^), and oxygen permeability (4.013 cm^3^·m^−2^·24 h^−1^·0.1MPa^−1^) were obtained under the addition of 1.5% TiO_2_-KH550. The tensile strength of TiO_2_-KH550 reinforced composite film was increased by 70% than that of the pure PVA/xylan composite film, and the water vapor and oxygen permeability were decreased by 31% and 41%, respectively. Moreover, the UV transmittance of the film at the wavelength of 400 nm was almost zero when adding ≈1.5~2.5% (weight ratio, based on the total weight of PVA and xylan) of TiO_2_-KH550, which indicated the PVA/xylan composite films were endowed with an excellent UV light shielding ability.

## 1. Introduction

Polysaccharide based materials with oxygen barrier properties show significant advantages over plastics when used as packaging materials [1]. Among them, xylan as a polar polymer has superior barrier properties to non-polar molecules (oxygen or aroma) [2,3]. Film materials made from xylan have great potential for food packaging [1,4]. The presence of hydroxyl groups of xylan facilitates the hydrogen bonding (intramolecular and intermolecular) during the film formation process, and the reaction of xylan with other polymers. However, hydroxyl groups also make xylan film hydrophilic [5], which leads to poor moisture barrier performance. Xylan has also been used to make composite films with other polymers, such as chitosan, cellulose, lignin, gluten, pectin, and polyvinyl alcohol (PVA).

PVA is a high molecular polymer with a linear structure. The PVA chain contains a large number of hydroxyl groups, resulting in a high hydrophilicity. The side groups -H and -OH of the PVA chain can enter the crystalline regions without producing stress due to the small volume. The higher the crystallinity, the lower the breathability of materials [6]. In addition, PVA also has good mechanical properties, heat resistance, resistance to organic solvents, resistance to organic contamination, and good film-forming properties [7]. Based on the superior properties of PVA, much work has been done on the preparation of composite films from PVA and natural polysaccharides (starch, chitosan, cellulose, hemicellulose, hyaluronic acid, and agar) for packaging applications. Our group has previously studied the effects of different additives (glycerol, urea, citric acid, BTCA, and TiO_2_ microparticles) on the PVA/xylan composite film properties [8,9,10,11].

Film materials prepared by compositing organic polymers and inorganic particles usually have better properties than those of organic films [12]. Generally, organic film material has high flexibility and low density, while inorganic particles have toughening effects. Introduction of inorganic particles into the organic polymer film can adjust the hydropathy balance of the film and impart the unique properties of inorganic particles to the composite film [13]. Hence, the polymer-inorganic compositing is an effective way to improve the performance of film materials.

Nanoparticles with good interfacial adhesion to polymers have a large specific surface area and high surface energy, and have been added into the polymer matrix to improve the properties [14]. Nano titanium dioxide (Nano-TiO_2_) has three crystal phase states of brookite, rutile, and anatase [15]. Rutile TiO_2_ shows excellent shielding performance against ultraviolet light due to its high scattering effect. Nano-TiO_2_ is widely used in air purification, wastewater treatment, and food packaging applications due to its non-toxicity, low cost, high stability, antibacterial ability, photocatalytic activity, and UV shielding effect [15,16,17,18,19,20,21]. Nano-TiO_2_ was also used in the PVA composite to increase its application characteristics [22,23,24].

Nanoparticles have a strong tendency to aggregate in water because of the high surface energy [23]. Once aggregated, the nanoparticles will have a much lower efficiency. Therefore, dispersants have to be added to improve the dispersion of nanoparticles in the medium. Inorganic phosphates can reduce the particle aggregation by changing the surface charge distribution like anticoagulants [25,26]. For instance, sodium hexametaphosphate (SHMP), an inorganic anionic surfactant, has been used for nanoparticle dispersion. Chemical modification of nanoparticles to reduce the surface energy is also an effective way to improve the dispersibility. Silane coupling agents are commonly used. They are amphoteric compounds with polar groups at one end of the molecule that can react with the hydroxyl groups of the nanoparticle, and the groups at the other end that can crosslink with organic polymers chemically or physically. They are widely used in the modification of composites, acting as a bridge between inorganic particles and organic polymers. Among them, γ-aminopropyltriethoxysilane (KH550) is often used in the modification of inorganic nanoparticles such as SiO_2_ and TiO_2_ [27,28].

The objective of our work was to develop PVA/xylan composite films with high mechanical strength and ultraviolet (UV) shielding ability. The effects of TiO_2_-KH550 particles on the mechanical properties, moisture and oxygen barrier properties, thermal stability, and UV shielding performance were investigated.

## 2. Materials and Methods

### 2.1. Materials

Beech wood xylan (*M_w_* of 130,000 g·mol^−1^) and polyvinyl alcohol (PVA, *M_w_* of 146,000–186,000 g·mol^−1^) were purchased from Sigma Aldrich (Karlsruhe, Germany). Nano-titanium dioxide (TiO_2_, Rutitle types, 98%), sodium hexametaphosphate (SHMP) and γ-aminopropyltriethoxysilane (KH550, 99%) were obtained from Macklin Reagent Company Limited (Shanghai, China). Lithium chloride (LiCl) and potassium nitrate (KNO_3_) were from Ke Miou reagent Company Limited (Tianjin, China). Deionized water was used for preparation of composite films. All materials were of analytical-reagent grade and used without further purification.

### 2.2. Synthesis of TiO_2_-KH550

**Figure 1:**
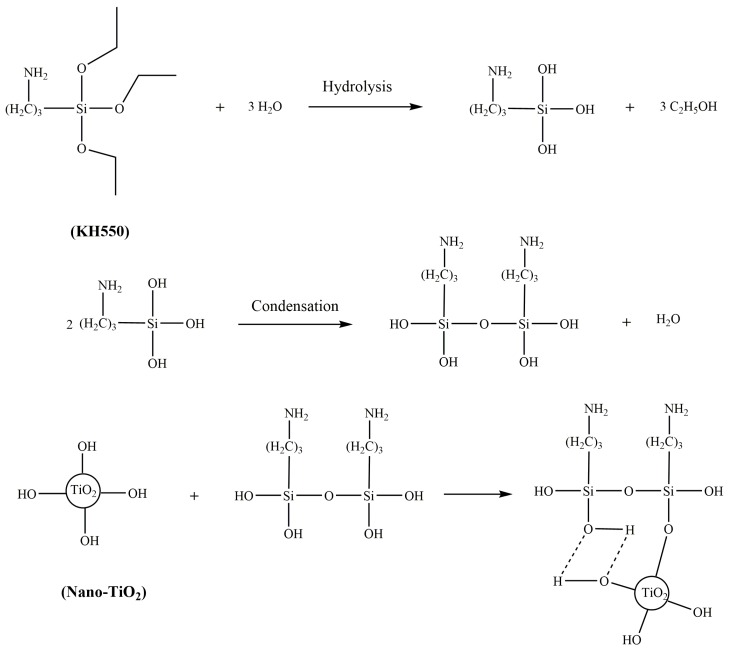
The synthesis of TiO_2_-KH550.

0.25 g of KH550 was added to deionized water and subjected to pre-hydrolysis under ultrasound treatment. After the pre-hydrolysis, 5 g of nano-TiO_2_ and 100 mL of ethanol were added and stirred at 80 °C for 5 h. Then, the above mixture was filtered and washed with ethanol and deionized water. Finally, the remaining solids (powder) were vacuum dried to constant weight to obtain the TiO_2_-KH550 powder [28]. The synthesis route was shown in Figure 1.

### 2.3. Characterization of TiO_2_-KH550

The Fourier transform infrared spectroscopy (FT-IR) spectrum of TiO_2_-KH550 was acquired by a Vertex 33 spectrometer (Bruker, Germany). The powder was dehydrated in an oven at 50 °C before analysis, and then mixed with KBr and pressed into a tablet for the measurement. 

Thermogravimetric analysis of TiO_2_-KH550 was performed using a thermal analyzer (TGA Q500, TA Instruments, New Castle, DE, USA). A total of 10 mg of dried samples were ground into fine powder and heated to 700 °C. The heating rate was kept at 10 °C min^−1^, and the nitrogen flow was maintained at 20 mL·min^−1^. 

A total of 0.1 g of TiO2-KH550 was added into the SHMP solution (0.04%) and sonicated for 10 min. The dispersion was allowed to stand in a 25 mL colorimetric tube for 24 h. After settling for another 24 h, a new batch of the supernatant was taken [29] and the final volume V (mL) was recorded. The gravity distribution index G of the dispersion is expressed by Equation (1):

G = (25 − V)/25(1)

A total of 0.1 g of TiO_2_-KH550 was added into SHMP solution (0.04%) and sonicated for 10 min. The dispersion was allowed to stand in a 25 mL colorimetric tube for 24 h. Then, 1 mL of the supernatant was taken and diluted to 50 mL with water, and the maximum absorbance was measured using an ultraviolet spectrometer (S3100, Scinco, Korea).

### 2.4. Preparation of TiO_2_-KH550 Reinforced PVA/xylan Composite Films

**Figure 2:**
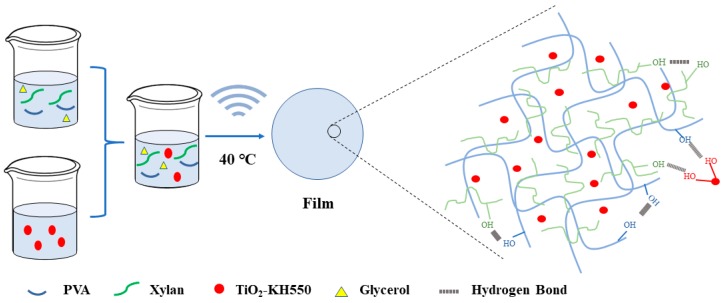
Synthesis process of composite films.

PVA was dissolved in 50 mL of 0.04% SHMP by heating to 95 °C. After cooling down to 80 °C, xylan and 10% glycerol (weight ratio, based on the total weight of PVA and xylan) were added. The effect of glycerol was to improve the compatibility of PVA/xylan. The weight ratio of PVA to xylan was 3. The 0~2.5% TiO_2_-KH550 (weight ratio, based on the total weight of PVA and xylan) was dispersed in 50 mL of 0.04% SHMP solution and stirred for 30 min, then sonicated for another 30 min. The TiO_2_-KH550 nanoparticle dispersion was gradually dropped into the PVA/xylan solution under stirring and the addition speed is five milliliters per minute. After an ultrasonic defoaming treatment, the above mixture was poured onto a polytetrafluoroethylene (PTFE) mould and dried in an oven at 40 °C, and the resulting composite films were obtained. All the films were kept at 23 °C with 50% relative humidity (RH) for at least 48 h prior to measurements. The composite films were named KT0, KT0.5, KT1, KT1.5, KT2, and KT2.5 according to the dosage of TiO_2_-KH550 nanoparticles (0~2.5%). The synthesis process of the composite films was proposed in Figure 2. 

### 2.5. Characterization of Composite Films

The mechanical properties of PVA/xylan composite films, such as tensile strength (TS) and elongation at break (EAB), were evaluated using a tensile testing machine (Instron 5565, Boston, MA, USA). 

The water vapor permeability value was measured according to the method described by Kayserilioglu et al. [30]. The water vapor permeability was calculated by Equation (2):

(2)  WVP=(Δm×L )/(A×t×Δp)

where WVP is water vapor permeability (g·m^−1^·s^−1^·Pa^−1^). Δm/t (g·s^−1^) is the gain rate of bottle. A (m^2^) is the exposed area of the films. L (m) is the mean thickness of the film. and Δp (Pa) is the difference in partial water vapor pressure between the two sides of film samples. 

The composite films were cut into 25 × 25 mm^2^ samples and dried to constant weight (m_0_, g). The dried sample was immersed in 50 mL deionized water at room temperature for 24 h. Then, the swollen film was dried again for 24 h at 50 °C for the final weight m_1_. The water solubility (WS) was calculated according to Equation (3):

(3) $$\;\mathrm{WS}=\left(m_{0}-m_{1}\;\right)/m_{0}\times 100\%$$

The oxygen permeability OP (cm^3^·m^−2^·24h^−1^·0.1MPa^−1^) was measured at 23 °C with RH 50% using a differential pressure gas permeation apparatus (Labthink VAC-V1, Jinan, China). The vacuum degassing was carried out for 8 h prior to the measurement. The oxygen pressure was kept at 0.5 MPa.

The composite films were cut into 25 × 25 mm^2^ samples and kept in a desiccator (using saturated LiCl solution to maintain a RH of 11%) until reaching a constant weight (m_2_, g). Then the sample was placed in another desiccator (using saturated KNO_3_ solution to maintain a RH of 95%) and weighed (m_i_, g) at intervals. Finally, the sample was dried to a constant weight (m_c_, g). The moisture absorption W was calculated according to Equations (4) and (5):

(4) $$\;W_{c}=\left(m_{2}-m_{c}\;\right)/m_{c}\times 100\%$$

(5) $$\;W_{i}\;=\left(m_{i}-m_{c}\;\right)/{\;m}_{c}\times 100\%$$

where W_c_ is the moisture absorption of the initial sample.

The thermal degradation properties were found using thermogravimetric analysis on a simultaneous thermal analyzer (TGA Q500, TA Instruments, New Castle, DE, USA). The specimen was heated from room temperature to 700 °C. The heating rate was kept at 10 °C·min^−1^, and the nitrogen flow was maintained at 20 mL· min^−1^. 

The ultraviolet light shielding performance was evaluated using an ultraviolet-visible spectrometer (Shimadzu UV1800, Tokyo, Japan), which was scanned in the range of 200–900 nm.

X-ray diffraction of TiO_2_-KH550 and PVA/xylan composite films were analyzed using an X-ray diffractometer (Bruker, Karlsruhe, Germany). The angle of diffraction (2θ) was varied from 5–80°. The scan step and scan speed were 0.02° and 0.1 s/step, respectively.

The surface and fracture surface morphology of the composite films were investigated using a scanning electron microscopy (Hitachi S3700, Tokyo, Japan). The test was operated in high vacuum mode at an acceleration voltage of 15 kV. 

The surface roughness of the films was measured by atomic force microscopy (AFM, Veeco DI Nanoscope 3a, New York, NY, USA) with tapping mode at room temperature in air. 

## 3. Results and Discussion

### 3.1. Characterizations of TiO_2_-KH550

**Figure 3:**
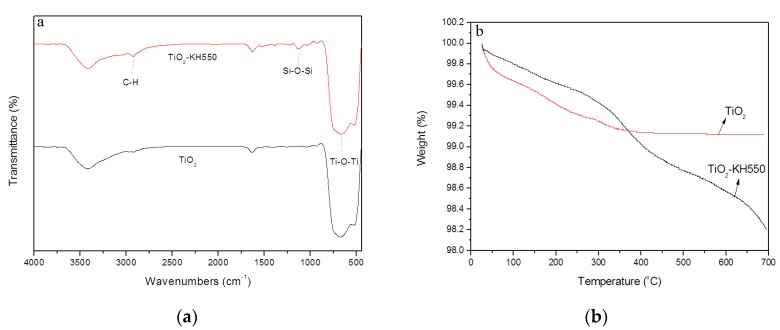
FTIR spectra (**a**) and TGA (**b**) of TiO_2_ and TiO_2_-KH550.

The FTIR spectra of TiO_2_ and TiO_2_-KH550 were shown in Figure 3a. In the spectra of TiO_2,_ the characteristic band at ≈480–800 cm^−1^ was assigned to the stretching vibration of Ti-O bond [31]. The characteristic band at 2930 cm^−1^ is assigned to the stretching vibration of CH_2_- [32]. The absorption band of Si–O–Si bond is observed at 1101 cm^−1^ in the spectra of TiO_2_-KH550 indicating the successful modification [33]. Furthermore, the intensity of the Ti–O bond and CH_2_- bond increased, which also implied that KH550 was successfully grafted onto TiO_2_.

Figure 3b shows the thermal stability of TiO_2_ and TiO_2_-KH550. The weight loss of TiO_2_ particles is mainly due to the moisture content in the sample. Below 200 °C, the weight loss of TiO_2_-KH550 is lower than that of TiO_2_. This is because the hydrophobic long chain is introduced onto TiO_2_, making TiO_2_-KH550 absorb less water [28]. At ≈320 °C~420 °C, the weight loss of TiO_2_-KH550 is mainly due to the dehydration of silanol groups, while the weight loss at 550 °C to 700 °C is due to the degradation of silane long chains [34]. The total weight loss of TiO_2_ and TiO_2_-KH550 at 700 °C were 1.8% and 0.88%, respectively. The grafting rate of KH550 is about 0.92 wt%.

**Figure 4:**
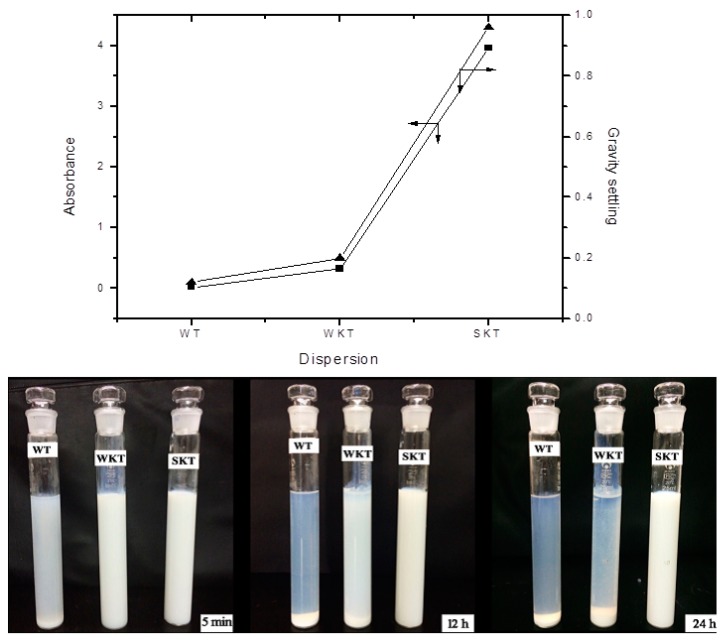
The absorbance and gravity distribution index of TiO_2_-KH550 dispersion (WT refers to the dispersion of nano-TiO_2_ in deionized water, and WKT refers to the dispersion of TiO_2_-KH550 in deionized water; SKT, the dispersion of TiO_2_-KH550 in 0.04% SHMP).

The TiO_2_ and TiO_2_-KH550 nanoparticles were dispersed in water and 0.04% SHMP solution, respectively, and the gravity stability and absorbance were determined as shown in Figure 4 (WT refers to the dispersion of nano-TiO_2_ in deionized water. WKT and SKT refer to the dispersion of TiO_2_-KH550 in deionized water and 0.04% SHMP, respectively). The greater the gravity distribution index (G) and absorbance, the better the dispersibility, and the higher the stability. The absorbance of the supernatant of the suspension is proportional to the dispersion of the powder and the stability of the suspension. Therefore, the change in absorbance could reflect the dispersion effect of nanomaterials [35]. When dispersed in water, the value of G and the absorbance of TiO_2_-KH550 were slightly higher than those of TiO_2_. When dispersed in 0.04% SHMP solution, the value of G and the absorbance of TiO_2_-KH550 particles were markedly increased compared to the particles in water. It can be seen from the macro photograph (Figure 4), no precipitation occurred in the three solutions after five minutes of standing. However, precipitation occurred in both solutions of WT and WKT after standing for 12 h, indicating the uneven dispersion. After standing for 24 h, the phenomenon is more obvious. Therefore, TiO_2_ modified by a KH550 coupling agent was dispersed in 0.04% SHMP solution to obtain a homogeneous dispersion.

### 3.2. Characterizations of Films

#### 3.2.1. Mechanical Properties

**Figure 5:**
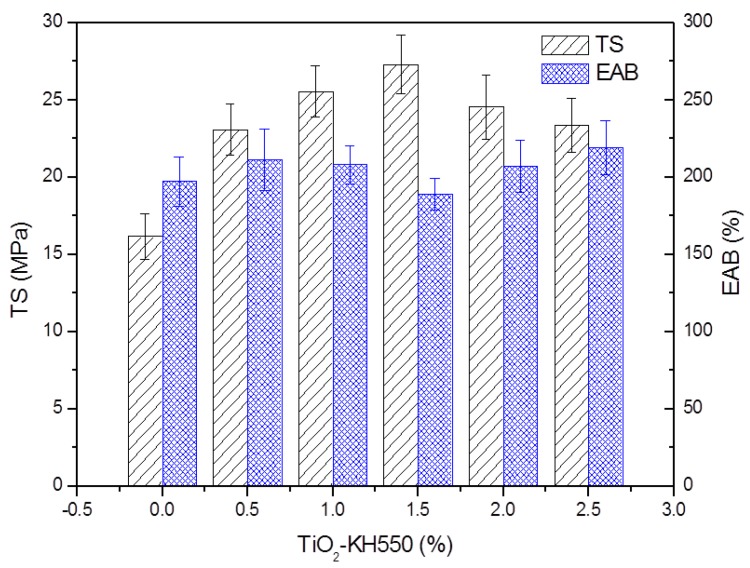
Mechanical properties of the composite films (KT0~KT2.5).

Figure 5 illustrates the effects of TiO_2_-KH550 dosage on the TS and EAB of the composite films. The addition of TiO_2_-KH550 led to a significant improvement in TS. The TS increased with the increasing dosage of TiO_2_-KH550 and then decreased. When the dosage was 1.5%, the TS reached a highest value (27.3 MPa). The high TS of composite films was attributed to the better dispersion of TiO_2_-KH550 and its interaction with the polymer matrix. However, when further increasing the dosage of TiO_2_-KH550, the TS decreased. At a high dosage, nanoparticles tended to aggregate to reduce the surface free energy, which led to a decrease in the hydrogen bonding between TiO_2_-KH550 and the polymers [22,23,24,25,26,27,28,29,30,31,32,33,34,35,36]. It should be noted that a strong interaction between TiO_2_-KH550 and PVA/xylan matrix affected the mobility of PVA and xylan molecules and decreased the EAB value of the composite film. However, under our experimental conditions, all the composite films had good flexibility and high EAB values (more than 189%). 

#### 3.2.2. Water Vapor Permeability, Solubility, and Oxygen Permeability

The water vapor permeability (WVP), water solubility (WS), and oxygen permeability (OP) of KT0~KT2.5 are shown in Table 1. The WVP and WS of TiO_2_-KH550-reinforced PVA/xylan composite films are lower than those of PVA/xylan films. This was due to the fact that TiO_2_-KH550 could enter the gap between PVA and xylan matrix and interact with the polymers to form a dense impermeable structure [37]. KT1.5 and KT1 have the lowest WVP and WS, respectively, indicating that TiO_2_-KH550 could be well dispersed in the polymer matrix at those dosages. Very low dosage (KT0.5) of TiO_2_-KH550 could possibly result in a high crystallinity, which made it difficult for moisture to enter the film [38,39].

In food packaging materials, a gas barrier is the key factor to prevent food spoilage [1]. As shown in Table 1, the OP of KT0 was higher than those of KT0.5~KT2.5, which indicated that the addition of nanoparticles improved the oxygen barrier properties of the composite films. When the dosage of TiO_2_-KH550 was at 1.5%, the OP reached the lowest (4.013 cm^3^·m^−2^·24h^−1^·0.1MPa^−1^). 

#### 3.2.3. Moisture Absorption

**Figure 6:**
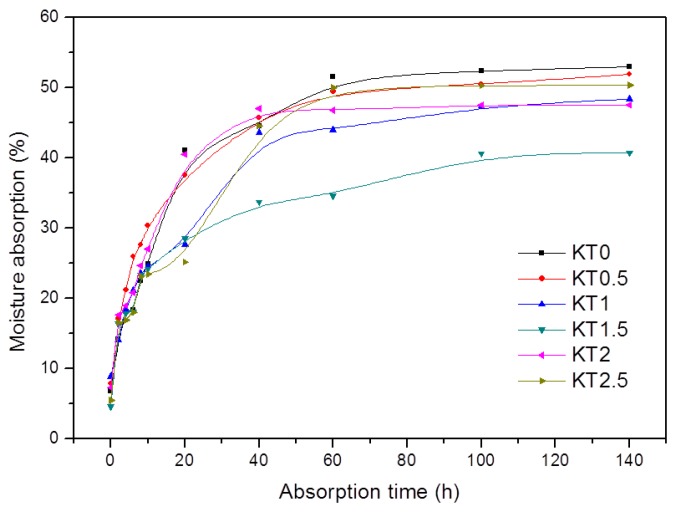
Moisture absorption of the composite films (KT0.5~KT2.5).

The moisture absorption of KT0~KT2.5 is shown in Figure 6. All the samples reached a hygroscopic equilibrium state after 60 h. The equilibrium moisture absorption of KT0 was larger than those of KT0.5~KT2.5. The moisture absorption of KT0.5 and KT2.5 were similar to that of the pure PVA/xylan film. It could be concluded that TiO_2_-KH550 improved the water tightness of the composite films. KT1.5 had the lowest moisture absorption (~40.7%), because TiO_2_-KH550 nanoparticles were evenly distributed in the film and strongly interacted with the polymers, forming a more compact film [40]. At the same time, under the condition of good dispersion and interaction, the voids inside the composite film were reduced, and the water vapor absorption storage space was small, so that the equilibrium moisture absorption was small. However, further increasing the dosage of TiO_2_-KH550 decreased the moisture absorption due to the steric hindrance effect of excessive nanoparticles. 

#### 3.2.4. Thermal Stability

**Figure 7:**
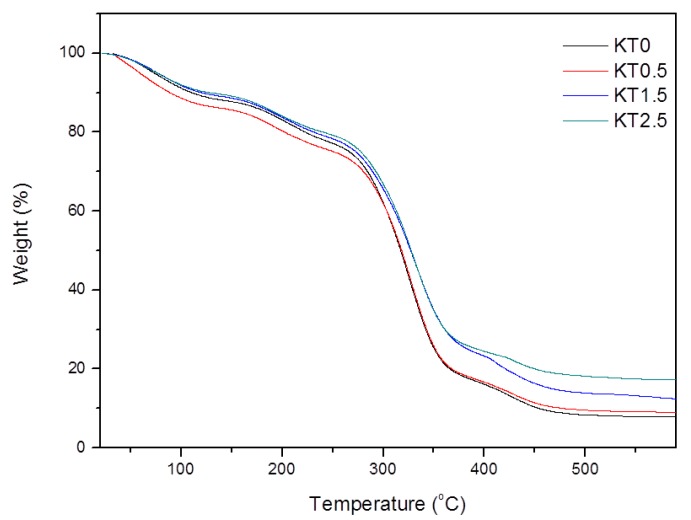
TGA of the composite films (KT0.5~KT2.5).

The thermal stability of KT0, KT0.5, KT1.5, and KT2.5 was illustrated in Figure 7. The weight loss of the composite films was divided into the following four stages: 30~120 °C, 120~220 °C, 220~380 °C, and 380~470 °C. The mass loss below 120 °C was due to the evaporation of water in the film, whereas the degradation of the glycerol small molecule occurred mainly in the range of 120~220 °C. The loss at about 220~380 °C was attributed to the degradation of the side chains of PVA and xylan. The weight loss above 380 °C was owing to the carbonation of the polymers. The temperature of the maximum degradation of KT1.5 and KT2.5 in the third and fourth stages were higher than that of KT0, which indicated that the former two had better thermal stability. This can be explained by the fact that excessive TiO_2_-KH550 slowed down the small-molecule gas transfer during the degradation process. Moreover, more interaction between TiO_2_-KH550 and the PVA/xylan matrix means more energy is needed for thermal degradation. Therefore, TiO_2_-KH550 nanoparticles was conducive to improving the thermal stability of the composite films [41]. 

#### 3.2.5. Ultraviolet Light Shielding Performance

**Figure 8:**
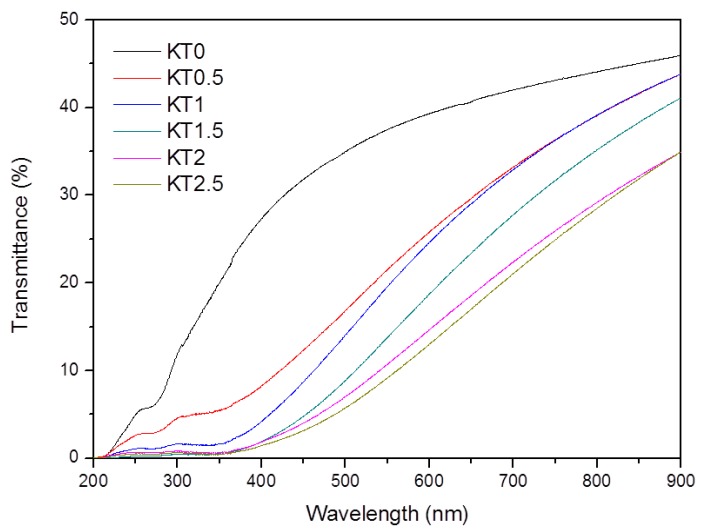
UV transmittance of the composite films (KT0.5~KT2.5).

Figure 8 illustrates the UV transmittance of KT0~KT2.5 films. Compared to the pure PVA/xylan composite film, the UV transmittance of the TiO_2_-KH550 reinforced composite films was significantly reduced. In particular, the UV transmittance of KT1.5, KT2, and KT2.5 were almost zero at the wavelengths below 400 nm. This is due to the high scattering effect of rutile Nano-TiO_2_ [42]. Our results were consistent with those of Li, Chiang, and Mallakpour et al [43,44,45]. Thus, the resulting PVA/xylan composite films with good UV shielding performance have great potential for food packaging. 

#### 3.2.6. XRD Analysis

**Figure 9:**
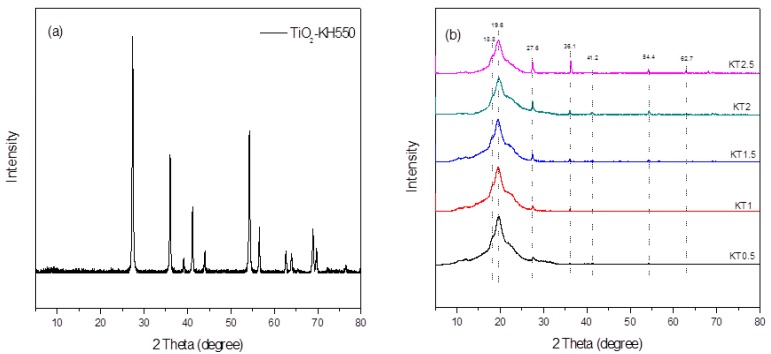
XRD of TiO_2_-KH550 (**a**) and the composite films (**b**).

Figure 9 exhibits the X-ray diffraction patterns of TiO_2_-KH550 and KT0.5~KT2.5 films. For TiO_2_-KH550, the characteristic peaks of rutile TiO_2_ were observed, indicating that the crystal form of the nano-particles has not changed obviously after modification. For KT0.5~KT2.5, the characteristic diffraction peaks of xylan at a 2θ = 18.8° [46] and of PVA at a 2θ = 19.4° [47] combined into a broad diffraction peak at a 2θ = 19.6° after forming the composite film, indicating that the crystalline area of xylan was changed during the formation of film. New diffraction peaks occurred at 27.6°, 36.1°, 41.2°, and 54.4°, which are the main characteristic peaks of rutile TiO_2_ particles. With the increase of TiO_2_-KH550 dosage, the characteristic peak intensities of the nanoparticles became much higher. Thus, the relative intensities of characteristic peaks of PVA and xylan was lowered by TiO_2_-KH550. Therefore, the crystallinity of the composite film was decreased with the increasing dosage of TiO_2_-KH550. 

#### 3.2.7. SEM Analysis

**Figure 10:**
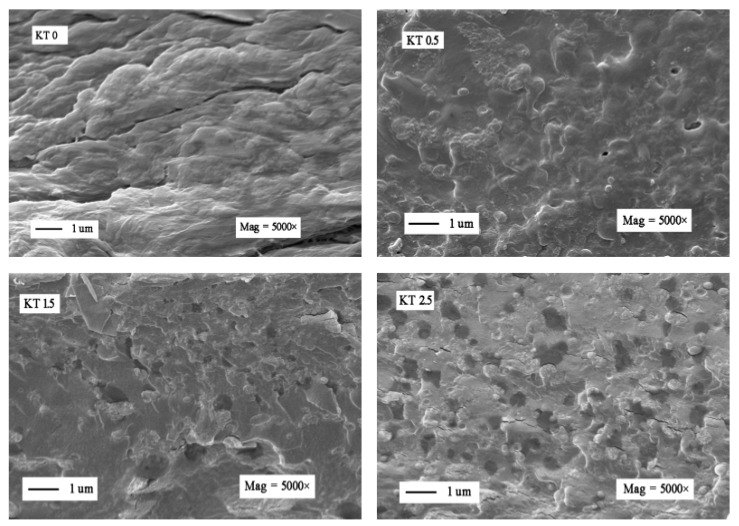
Cross-sectional SEM images of the composite films (KT0, KT0.5, KT1.5, and KT2.5).

The SEM micrographs of KT0, KT0.5, KT1.5, and KT2.5 films are shown in Figure 10. The TiO_2_-KH550 particles distributed in the polymer matrix were observed on the cross section images of KT0.5~KT2.5. No obvious particle separation phenomenon was seen on the cross section images of KT0.5~KT2.5 films, suggesting the good compatibility between the TiO_2_-KH550 particles and the polymer matrix. The cross-section micrographs could be described with regard to the degree of interactions and miscibility of different components [9], from which more interspace was observed in the KT2.5 film compared to other films. The KT0.5 and KT1.5 films had a better uniform structure owing to the better dispersion of TiO_2_-KH550. 

#### 3.2.8. AFM Analysis

**Figure 11:**
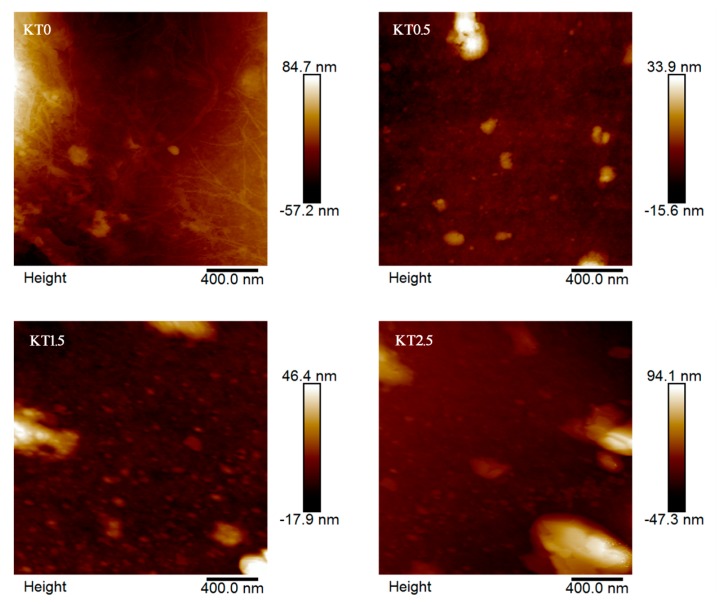
AFM images of the composite films (KT0, KT0.5, KT1.5, and KT2.5).

Figure 11 shows the AFM images of KT0, KT0.5, KT1.5, and KT2.5 films. After adding TiO_2_-KH550, the surface of the composite films become more rough with some protrusions owing to the encapsulation effect of TiO_2_-KH550 by PVA and xylan [48]. Moreover, with the increase of the TiO_2_-KH550 dosage, the surface roughness of the composite film was increased. The average surface roughness of KT0, KT0.5, KT1.5, and KT2.5 films were 1.75, 2.74, 4.15, and 12.0 nm, respectively. The composite films changed to become coarse because the nanoparticles (TiO_2_-KH550) remained adhered to the composite films. As the dosage of TiO_2_-KH550 increased, the more adhering nanoparticles, the more rough the surface became.

## 4. Conclusions

In this paper, TiO_2_ was modified to prepare TiO_2_-KH550, and the TiO_2_-KH550 could be well dispersed in the SHMP solution (0.04%) under ultrasonic treatment. In addition, a series of TiO_2_-KH550-reinforced PVA/xylan composite films were prepared. The composite films had high UV shielding efficiency and excellent mechanical properties under the addition of TiO_2_-KH550. When the dosage of TiO2-KH550 was 1.5%, the TS (tensile strength) of the composite film reached the highest (27.3 MPa), which was 70% higher than that of a pure PVA/xylan composite film. TiO_2_-KH550 could be well dispersed in SHMP solution (0.04%) under ultrasonic treatment. And, the composite films had the lowest WVP (2.75 × 10^−11^ g·m^−1^·s^−1^·Pa^−1^), OP (4.013 cm^3^·m^−2^·24 h^−1^·0.1 MPa^−1^), and W (40.7%) at the dosage of 1.5% TiO2-KH550. TiO_2_-KH550 could also improve the thermal stability and UV shielding efficiency of the composite films. In general, when the dosage of TiO_2_-KH550 was 1.5%, the properties of the composite film is optimal.

## Figures and Tables

**Table 1:** The water vapor permeability, solubility, and oxygen permeability of the composite films (KT0.5~KT2.5).

Samples	WS (%)	WVP (10^−11^ g·s^−1^·m^−1^·Pa^−1^)	OP (cm^3^·m^−2^·24 h^−1^·0.1MPa^−1^)
KT0	32.38 ± 2.3	3.97 ± 0.8	6.823
KT0.5	29.93 ± 1.8	3.25 ± 0.3	5.372
KT1	27.72 ± 1.7	3.57 ± 0.6	4.092
KT1.5	28.84 ± 2.3	2.75 ± 0.3	4.013
KT2	29.78 ± 2.1	3.43 ± 0.5	6.138
KT2.5	30.82 ± 2.6	3.61 ± 0.5	6.068
